# Clinical and Therapeutic Profile of Non-Hodgkin’s Lymphoma: A Retrospective Study From a Najran Oncology Center

**DOI:** 10.7759/cureus.40125

**Published:** 2023-06-08

**Authors:** Ahmed M Badheeb, Faisal Ahmed, Musadag Elhadi, Nasher Alyami, Mohamed A Badheeb

**Affiliations:** 1 Oncology, King Khalid Hospital-Oncology Center, Najran, SAU; 2 Urology, Ibb University, Ibb, YEM; 3 Medicine, King Khalid Hospital, Najran, SAU; 4 General Medicine, Ministry of Health Holdings, Najran, SAU; 5 Hematology, Maternity and childern Hospital, Najran, SAU; 6 General Medicine, King Khalid Hospital, Najran, SAU

**Keywords:** chemotherapy, saudi arabia, najran city, non-hodgkin’s lymphoma, survival rate

## Abstract

Background

Non-Hodgkin lymphomas (NHL) represent a group of lymphoproliferative disorders, with a limited understanding of their clinical spectrum, primary extranodal variety, histopathology, and immunohistochemistry, particularly in developing countries. The objective of this study was to evaluate the clinicopathological characteristics and survival rates of NHL patients treated at King Khaled Hospital in Najran City, Saudi Arabia.

Method

In this retrospective chart review of NHL cases that received chemotherapy at the Oncology Center of King Khaled Hospital in Najran City, Saudi Arabia, between 2014 and 2021, we evaluated the clinicopathological features, survival rate, and associated factors. Using standardized data collection sheets, we extracted information on patients' age, gender, tumor type, stage, baseline laboratory evaluations, disease status, cancer treatment, and survival from electronic medical records. Univariate analysis was employed to identify factors associated with mortality and relapse.

Results

We included 43 NHL patients with a mean age of 59.23 ± 20.17 years, with a higher frequency among females (65.1%). B symptoms were present in 32 (74.4%) cases. The common primary site was peripheral lymph nodes (79.1%). Diffuse large B-cell lymphoma was the most common morphologic type (67.4%), and 46.5% of the patients had advanced-stage disease (stages III-IV). All patients received the first line of treatment, with the most common chemotherapy used being the RCHOP regimen (67.4%). Additionally, radiotherapy was performed in seven (16.3%) cases. Relapse occurred in eight (18.6%) cases with a median period of 47.5 months (Min: 20 - Max: 77 months). The mean overall survival time was 43.25 ± 2.98 months (range 12-168 months), and the one, three, and five-year survival rates were 91%, 58%, and 38%, respectively and the mortality rate was 32.6%. Univariate analysis showed that Burkitt lymphoma had (odds ratio (OR): 11.87; 95% confidence interval (CI): 1.58-89.09, p=0.016) and elevated lactate dehydrogenase (LDH) ((OR: 1.26; 95% CI: 0.35-4.54), p=0.014) were associated with mortality. Moreover, advanced age and the total number of first chemotherapy cycles were associated with relapse (p< 0.05).

Conclusion

The study highlights the variability of NHL cases, with a significant proportion presenting with advanced-stage disease and in middle age. The results suggest poor survival rates for patients with Burkitt lymphoma subtypes and elevated LDH levels.

## Introduction

Lymphomas represent a diverse array of malignancies of a lymphoid origin. Despite the advancement in the pathobiological understanding of their origin, lineage, and precursors, there is a controversy in the optimal classification of lymphomas [1]. International Consensus Classification (ICC) and the World Health Organization classification are primarily based on clinical, morphological, and immune-phenotypical characteristics [2]. Regardless, lymphoma is broadly classified into Hodgkin lymphoma (HL) and non-Hodgkin lymphoma (NHL), the latter accounts for more than 85% of cases, and more than 90% of these cases could be classified as mature B cells NHL [3]. In the United States, NHL accounts for 4% of all malignancies, and in 2023, an estimated 80,550 persons (44,880 men and 35,670 women) would be diagnosed with NHL [4]. The International Agency for Research on Cancer has calculated an age-standardized incidence rate of NHL of 6.5 per 100,000 male patients, with an age-standardized mortality rate of 4.3 per 100,000 male patients [5]. Furthermore, the prevalence of this has increased significantly throughout the year, particularly in Saudi Arabia. In 2008, NHL was one of the most common kinds of cancer in Saudi Arabia, ranking second in cancer incidence among men (male to female ratio = 122:100) [6]. Although not fully understood, the pathogenesis of NHL is believed to be multifactorial, including infectious, or environmental exposure (e.g., chemical), immunodeficiencies (e.g., AIDS), autoimmune disease, and genetic aberrations [7].

The epidemiological variation of lymphoma is not consistent in the literature in the Kingdom of Saudi Arabia [8,9]. Nevertheless, malignant lymphoma remains a highly prevalent malignancy in KSA with increased reported cases among younger people. The prevalence of NHL was reported to be 6.4% of all new lymphoma cases diagnosed in 2014 [10]. Despite the limited available literature, NHL exhibits diverse clinical manifestations and pathologies, emphasizing the crucial need for establishing a comprehensive management framework. [7]. This study aims to review the clinical and histopathological characteristics, possible risk factors, and survival of NHL over seven years in the Najran region, Saudi Arabia.

## Materials and methods

Study design

A retrospective cross-sectional chart review study was conducted in the oncology center of King Khaled Hospital, Najran City including NHL cancer patients receiving chemotherapy to investigate the clinicopathological characteristics of NHL patients, and survival rates and associated factors in the period between 2014 and 2021. The Ethics Research Committees of King Khaled Hospital provided their approval for the study (ID: 2022-44 E, on September 4, 2022), which adhered to the ethical principles outlined in the Declaration of Helsinki. The samples represent full coverage.

Inclusion criteria

All adult patients (aged 18 years or older) diagnosed to have NHL in King Khaled Hospital, Najran City, Saudi Arabia, over seven years (2014 to 2021) were included in the study.

Exclusion criteria

Patients who missed follow-ups and those who had surgical procedures were excluded.

Data collection and definition

Using electronic medical records, we collected and analyzed data regarding age, gender, nationality, presence of B symptoms, tumor classification, histopathological evaluation, extent and sites of the disease (nodal/extranodal), staging, COVID-19 infection, baseline laboratory evaluation (white blood cell (WBC), hemoglobulin, albumin, lactate dehydrogenase (LDH), erythrocyte sedimentation rate (ESR), lymphocyte count, and platelet), management approach (first line, second line, salvage chemotherapy administration, and radiotherapy), response to treatment (relapse or remission), current status (survive, or died), and overall survival (OC).

The diagnosis of NHL was confirmed through histological examination of a lymph node biopsy/tissue and immunohistochemical stains, following the World Health Organization (WHO) classification [11]. We defined relapsed disease as a recurrence of the disease after varying periods of complete remission following first-line therapy [12]. The Ann Arbor classification was used to determine the clinical stage [13]. Stage 1 involves only one node or a group of contiguous nodes, whereas stage 2 involves two or more groups of nodes on the same side of the diaphragm. Stages 3 and 4 are both advanced, with nodes on both sides of the diaphragm or additional non-contiguous extra lymphatic involvement. The lymph nodes, spleen, thymus, and Waldeyer's ring are considered in the nodal localization, whereas additional organs are considered in the extranodal localization [14].

Statistical analysis

Mean and standard deviation was used to report quantitative data, while frequencies and percentages were used for qualitative variables. The normality of the data was confirmed using the Smirnov-Kolmogorov test. Independent samples T-test or Mann-Whitney U test were employed for quantitative variables, while Chi-square or Fisher's exact test was used for qualitative variables. Kaplan-Meier test was used to calculate overall and event-free survival curves. The mean OS was calculated from diagnosis to death from any cause. Univariate analysis was conducted to identify independent risk factors associated with survival, with effect size reported using odds ratio (OR) and confidence interval (CI) set at 59%. Statistical significance was set at p < 0.05. Statistical analysis was carried out using IBM SPSS version 22 software (IBM Corp., Armonk, NY).

## Results

Characteristics of the patients

Of 43 NHL patients, the mean age was 59.23 ± 20.17 years, and most of them 28 (65.1%) were female. B symptoms were presented in 32 (74.4%) cases. Peripheral lymph nodes were the common primary site (79.1%), diffuse large B-cell lymphoma was the commonly morphologic type (67.4%), and 46.5%of the patients presenting with advanced stage disease (stages III-IV). Radiotherapy was performed in seven (16.3%) cases. COVID-19 infection was documented in five (11.6%) cases. The socio-demographic characteristics of patients were summarized in Table 1.

**Table 1:** Baseline patients’ characteristics. Abbreviation: COVID-19: Coronavirus disease of 2019, RCVP: Rituximab with cyclophosphamide, vincristine, and prednisone, RCHOP: Rituximab with cyclophosphamide, doxorubicin, vincristine and prednisone, Hyper CVAD: Hyper-fractionated cyclophosphamide, vincristine, adriamycin, and dexamethasone.

Variables	N (%)
Age (year), mean ± SD	59.23± 20.17
Gender	
Male	15 (34.9)
Female	28 (65.1)
Nationality	
Saudi	20 (46.5)
Non-Saudi	23 (53.5)
Primary site	
Peripheral lymph nodes	34 (79.1)
Others	9 (20.9)
Immunophenotype (B cell)	100%
Pathologic finding	
Follicular lymphoma	6 (18.7)
Diffuse large B cell lymphoma	29 (67.4)
Burkitt lymphoma	2 (4.7)
Lymphoblastic lymphoma	1 (2.3)
Nodal marginal zone lymphoma	2 (4.7)
Mantle Cell lymphoma	1 (2.3)
Stage	
Early (I, II)	23 (53.5)
Advanced (III, IV)	20 (46.5)
B symptoms	32 (74.4)
Chemotherapy type	
RCVP	8 (18.6)
RCHOP	29 (67.4)
R-CHOP/R-CVP	1 (2.3)
Hyper CVAD	5 (11.6)
Radiotherapy	7 (16.3)
COVID infection	5 (11.6)
Relapse	8 (18.6)
Death	14 (32.6)
Survival (Months), (mean ± SD)	43.25 ± 2.98 (range 12-168)

Laboratory data

Hemoglobulin level was normal (more than 12 g/dL) in 14 (32.6%) cases, WBC was normal (4.5 to 11.0 × 10^9^/L) in 38 (88.4%) cases, the ESR was >50 (mm/hour) in 10 (23.3%) cases, and the LDH (U/L) was less than 320 U/L in 20 (46.5%) cases and high in 23 (53.5%) cases (Table 2).

**Table 2:** The laboratory data.

Variables	N (%)
Hemoglobulin (g/dL)	
Normal (More than 12)	14 (32.6)
Mild (>10 <12)	17 (39.5)
Moderate (10-8)	8 (18.6)
Severe (8-6.5)	3 (7.0)
Life-threatening < 6.5	1 (2.3)
White blood cell	
Normal (4.5 to 11.0 × 10^9^/L)	38 (88.4)
High	5 (11.6)
Platelet (per microliter)	
Normal (150,000 - 400,000)	40 (93.0)
Higher than 400.000	3 (7.0)
lymphocyte count/mcL	
Normal	34 (79.1)
< 600	9 (20.9)
Erythrocyte sedimentation rate	
>50 (mm/hour)	10 (23.3)
Less than 50 (mm/hour)	33 (76.7)
Lactate dehydrogenase (U/L)	
Less 320	20 (46.5)
More than 320	23 (53.5)
Albumin (g/dL)	
Less than 3	5 (11.6)
More than 3	38 (88.4)

Response to therapy

The treatment approach was individualized using different regimens as first-line therapy. Our patients received various first-line therapies, including RCVP, RCHOP, R-CHOP/R-CVP, and Hyper CVAD in eight (18.6%), 29 (67.4%), one (2.3%), and five (11.6%) cases, respectively. Eight (18.6%) cases experienced relapse and were given second line (salvage) therapy. The mean time to relapse was 44.13 ± 22.23 months, with a median period of maintaining remission for 47 months (range: 20-77 months). Salvage therapy (high-dose chemotherapy) was effective in five cases, while three patients required third-line chemotherapy within 10 months.

Survival summary

**Figure 1:**
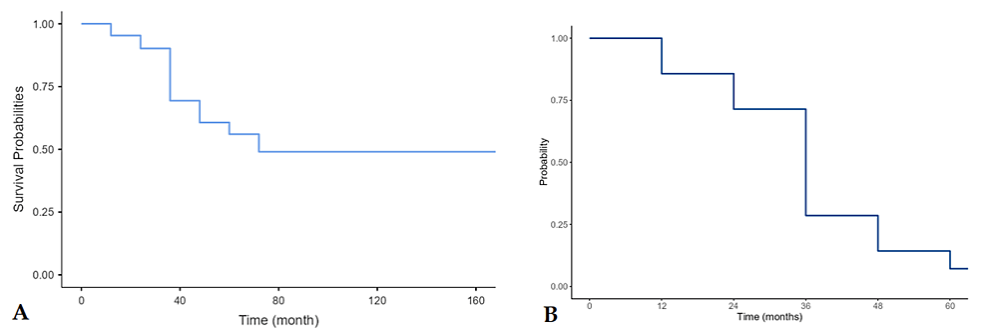
(A) Five-year overall survival. (B) Five-year event-free survival of NHL patients.

The overall mean survival time was 43.25 ± 2.98 months (range 12-168 months) and the mortality rate was 32.6%. The median survival was 60 months (95% CI: 36-72 months). The one, three, and five-year survival rate were 91% (95% CI: 82%-100%), 58% (95% CI: 45%-76%), and 38% (95% CI: 24%-60%) (Figures 1A, 1B).

Factors associated with survival

**Figure 2:**
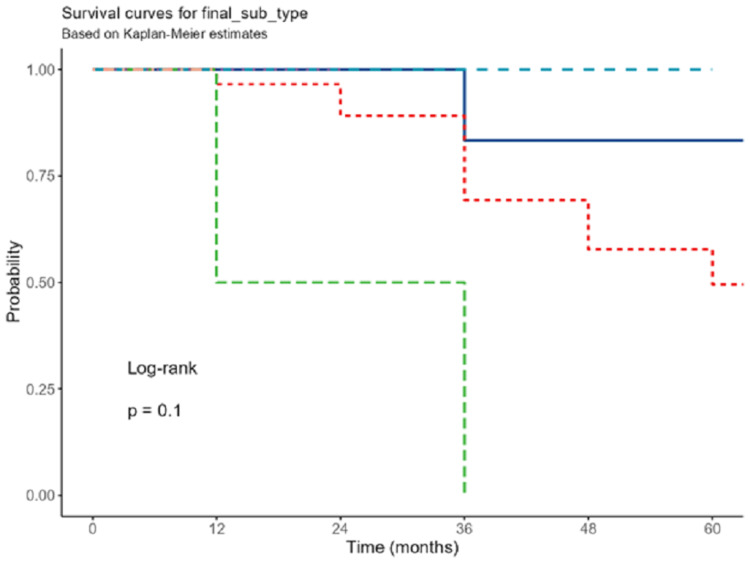
Survival in subtype groups.

In the univariate analysis, Burkitt lymphoma subtype (Figure 2) and elevated levels of LDH had an increased risk for mortality with (OR: 11.87; 95% CI: 1.58-89.09; p=0.016) and (OR: 1.26; 95% CI: 0.35-4.54; p=0.014), respectively (Table 3).

**Table 3:** Prognostic factors of overall survival. Abbreviations: LDH: Lactate dehydrogenase, ESR: Erythrocyte sedimentation rate, NL: lymph nodes lymphoma, LL: lymphoblastic lymphoma, FL: Follicular lymphoma, DLBCL: Diffuse large B-cell lymphoma, BL: Burkitt lymphoma, LL: Lymphoblastic lymphoma, NMZL: Nodal marginal zone lymphoma.

Variables	Subgroups	Mean (SD) or N (%)	Univariable	P-value
			HR (95%IC)	
Age (Year)	Mean ± SD	59.2±20.2	1.01 (0.99-1.04)	0.351
Nationality	Non- Saudi	20 (46.5)	Reference group	0.533
	Saudi	23 (53.5)	0.78 (0.37-1.65)	
Gender	Female	28 (65.1)	Reference group	0.298
	Male	15 (34.9)	1.76 (0.61-5.12)	
Primary site	Non-NL	9 (20.9)	Reference group	0.877
	NL	34 (79.1)	1.13 (0.25-5.06)	
Pathologic finding	FL	8 (18.6)	Reference group	-
	DLBL	29 (67.4)	1.56 (0.34-7.12)	0.568
	BL	2 (4.7)	11.87 (1.58-89.09)	0.016
	LL	1 (2.3)	1.35 (0.16-11.54)	0.999
	NMZL	2 (4.7)	9.13 (1.64-50.68)	0.999
	Mantle Cell	1 (2.3)	29.97 (2.57-348.95)	0.999
Stage	Early	23(53.5)	Reference group	0.855
	Advanced	20(46.5)	1.10 (0.39-3.15)	
B symptoms	No	11 (25.6)	Reference group	0.059
	Yes	32 (74.4)	0.44 (0.19-1.03)	
lymphocyte count (1 µL)	Normal	34 (79.1)	Reference group	0.724
	< 600	9 (20.9)	1.26 (0.35-4.54)	
ESR (mm/hr)	≥50	10 (23.3)	Reference group	0.849
	<50	33 (76.7)	1.13 (0.31-4.11)	
LDH (U/L)	<320	20(46.5)	Reference group	0.014
	≥320	23(53.5)	0.15(0.03-0.68)	
Radiotherapy	No	36 (83.7)	Reference group	0.173
	Yes	7 (16.3)	0.24 (0.03-1.86)	
COVID-19	No	38 (88.4)	Reference group	0.403
	Yes	5 (11.6)	0.42 (0.05-3.22)	
Relapse	No	35 (81.4)	Reference group	0.566
	Yes	8 (18.6)	1.41 (0.44-4.50)	

**Figure 3:**
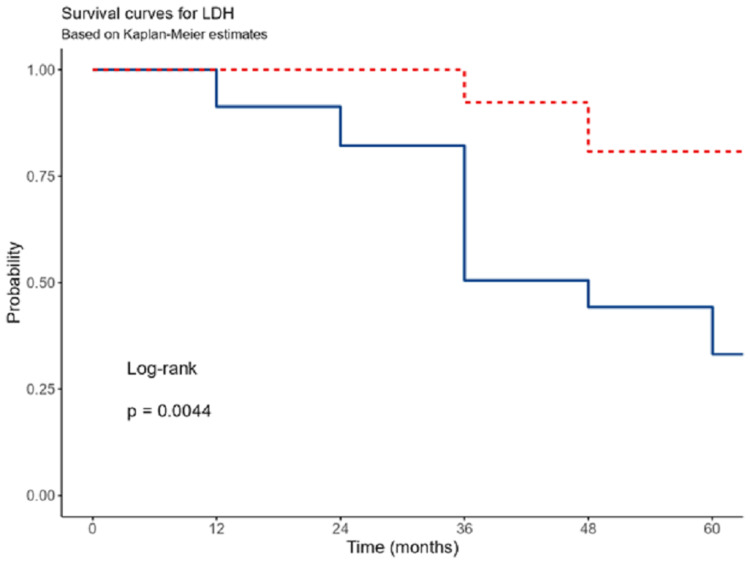
Survival regarding LDH level.

The hazard increased by a factor of (OR:1.01; 95% CI: 0.99-1.04) with an increase of 1 unit in age, but this was not statistically significant (p=0.351). The advanced stage was associated with higher mortality (OR: 1.19; 95% CI: 0.42-3.41), but this association was not statistically significant (p=0.744). When LDH levels were >320 U/L, the one-, three-, and five-year survival rates were 91.3% (95% CI: 80.5%-100%), 50.6% (95% CI: 31.6%-81%), and 37.9% (95% CI: 20.4%-70%), respectively. Conversely, when LDH levels were less than 320 U/L, the one-, three-, and five-year survival rates were 100.0% (95% CI: 100.0%-100%), 92.3% (95% CI: 78.9%-100%), and 80.8% (95% CI: 59.5%-100%), respectively. The difference between these survival rates was statistically significant (p=0.014) (Figure 3).

**Figure 4:**
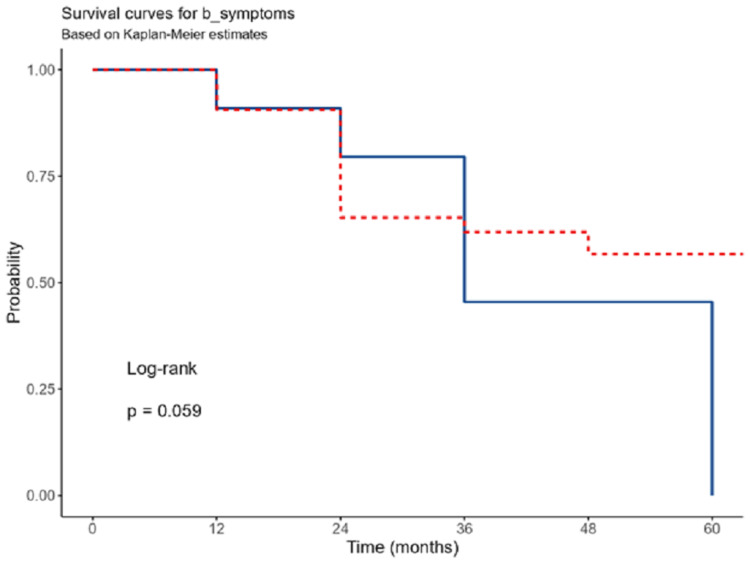
Survival regarding B symptoms presentations.

B-symptom presentation was associated with longer survival, with (OR: 0.40; 95% CI: 0.17-0.97), However, this association was not statistically significant (p=0.059) (Figure 4).

Factors associated with relapse

The univariate analysis of the initial presentation revealed significant correlations between certain factors and the relapsed group (Table 4). Specifically, advanced age and the number of chemotherapy cycles were significantly correlated with relapse (P=0.008 and 0.029, respectively). However, other clinical factors did not demonstrate any significant correlation with relapse.

**Table 4:** Factors affecting the relapse. Abbreviation: RCVP: Rituximab with cyclophosphamide, vincristine, and prednisone, RCHOP: Rituximab with cyclophosphamide, doxorubicin, vincristine and prednisone, Hyper CVAD: Hyper-fractionated cyclophosphamide, vincristine, adriamycin, and dexamethasone.

Variables	Subgroup	Total	No relapse	Relapse	P-value
Age	Mean (SD)	59.2 (20.2)	55.4 (19.4)	76.0 (14.8)	0.008
Nationality	Non-Saudi	20 (46.5)	18 (51.4)	2 (25.0)	0.250
	Saudi	23 (53.5)	17 (48.6)	6 (75.0)	
Gender	Female	28 (65.1)	23 (65.7)	5 (62.5)	1.000
	Male	15 (34.9)	12 (34.3)	3 (37.5)	
Primary Site	Others	2 (4.7)	2 (5.7)	0 (0.0)	0.852
	LN	34 (79.1)	26 (74.3)	8 (100.0)	
	GI	4 (9.3)	4 (11.4)	0 (0.0)	
	Nasopharynx	3 (7.0)	3 (8.6)	0 (0.0)	
Final Subtype	Fl	8 (18.6)	8 (22.9)	0 (0.0)	0.251
	DLBL	29 (67.4)	22 (62.9)	7 (87.5)	
	Burkitt Lymphoma	2 (4.7)	2 (5.7)	0 (0.0)	
	Lymphoblastic Lymphoma	1 (2.3)	1 (2.9)	0 (0.0)	
	NMZL	2 (4.7)	2 (5.7)	0 (0.0)	
	Mantle Cell	1 (2.3)	0 (0.0)	1 (12.5)	
B Symptoms	No	11 (25.6)	11 (31.4)	0 (0.0)	0.090
	Yes	32 (74.4)	24 (68.6)	8 (100.0)	
LDH	>320	23 (53.5)	18 (51.4)	5 (62.5)	0.704
	< 320	20 (46.5)	17 (48.6)	3 (37.5)	
ESR	< 50	33 (76.7)	25 (71.4)	8 (100.0)	0.165
	≥320	10 (23.3)	10 (28.6)	0 (0.0)	
Stage	Low	23 (53.5)	20 (57.1)	3 (37.5)	0.440
	High	20 (46.5)	15 (42.9)	5 (62.5)	
Chemotherapy Cycle Number	Mean (SD)	5.6 (3.4)	5.0 (3.2)	7.9 (3.0)	0.029
Chemo type	RCVP	12 (27.9)	11 (31.4)	1 (12.5)	0.285
	RCHOP	29 (67.4)	23 (65.7)	6 (75.0)	
	R-Bendamusine	1 (2.3)	0 (0.0)	1 (12.5)	
	Hyper CVAD	1 (2.3)	1 (2.9)	0 (0.0)	
Radiotherapy	No	36 (83.7)	30 (85.7)	6 (75.0)	0.597
	Yes	7 (16.3)	5 (14.3)	2 (25.0)	

## Discussion

NHL refers to a group of lymphoid neoplasms with varying clinical and biological characteristics. Our study presents data from a single institution in Najran City, Saudi Arabia on the survival and epidemiological trends of NHL. Our results showed that 47.1% of patients were middle-aged (39-59 years), and univariate analysis revealed that older age was associated with lower survival. This finding is consistent with a study conducted by Altowairqi et al. in Riyadh, Saudi Arabia, where 76% of patients were under 60 years of age, and advanced age was found to be a predictor of poor survival. [7]. Nevertheless, it is important to acknowledge that treatment outcomes can be influenced by a multitude of factors. One such factor is the age of the patient, as older individuals have been observed to exhibit lower tolerance to higher dosages of chemotherapy. However, it is noteworthy that the incidence of low-grade NHL is significantly higher among the elderly population as compared to their younger counterparts [15]. The younger age at which NHL was diagnosed in our study could be attributed to the high proportion of young individuals in Saudi Arabia or unique biological differences that require further investigation. On the other hand, the diagnosis of advanced-stage disease may be due to various factors, such as patients' delay in seeking medical attention due to inadequate knowledge or underestimation of symptoms, visiting several institutions before receiving a correct diagnosis, or the aggressive nature of the lymphoma subtype.

Previous reports have mentioned a slight male predominance [7,16]. In contrast, in this study, the most of patients were female (65.1%). This discrepancy may attribute to the low sample size of our patients.

In this study, diffuse large B-cell lymphoma was the most common type (67.4%), and 46.5% of patients presented in an advanced stage. A similar pattern of the advanced-disease stage at presentation and the most common morphological type of diffuse large B-cell lymphoma were observed in previous studies [7,16,17].

The prognostic factors for NHL are still not fully agreed upon, due to the inconsistent outcomes resulting from small retrospective studies. This lack of consensus makes it difficult to predict outcomes in these cases. However, some studies have identified certain factors that contribute to better outcomes in NHL patients. For instance, according to a study conducted by Huang et al., patients with germinal center B-cell-like subtype who underwent R-CHOP-like treatment and met certain criteria had longer OS and progression-free survival (PFS). These criteria included the lack of B symptoms, Ann Arbor stages I-II, ≤ 1 extranodal involvement, an Eastern Cooperative Oncology Group (ECOG) score of ≤ 1, and normal levels of LDH and β2 microglobulin levels. Additionally, the lower International Prognostic Index (IPI) or National Comprehensive Cancer Network-International Prognostic Index (NCCN-IPI) scores were identified as favorable prognostic factors for OS and PFS [18]. he NCCN-IPI was identified as a superior prognostic tool compared to the IPI for classifying NHL patients into groups with different risk levels. This improved performance can be attributed to the NCCN-IPI's allocation of higher points to negative risk factors, enabling better differentiation between patient groups. Additionally, the NCCN-IPI was observed to have better predictive capabilities in terms of clinical outcomes when using PFS as the endpoint [19]. Other factors included germinal center B-cell-like subtype, and R-CHOP-like treatment [18]. Another study reported favorable outcomes in patients with limited disease who were treated with radiation therapy [20]. Conversely, Altowairqi et al. reported poorer outcomes in older patients (≥60 years), those with higher LDH, and those with advanced-stage disease [7]. Another study found that lower complete response rates and higher relapse rates contributed to an increased risk of death. The study introduced two indexes, known as the international index and age-adjusted international index, which were more precise in predicting long-term survival than the Ann Arbor classification [21]. Furthermore, patients with poor performance status were found to have a higher treatment-related death rate [22]. Similar results were reported in a randomized study of patients with aggressive lymphoma in stages I-II, which found a low OS rate of 48% for those with two or more adverse risk factors. These risk factors included being 0 years of age or older, stage II disease, elevated serum LDH levels, and a performance status of 2 or higher [23].

Our findings suggest that the presence of B symptoms may be associated with survival in NHL patients, although this association did not reach statistical significance in our study. The reasons for the higher percentage of B symptoms in our cohort remain unclear. These findings are consistent with earlier studies by Bateganya et al. and Kane et al., which also reported on the potential impact of B symptoms on NHL outcomes [24,25]. It is important to note that the studies referenced in this discussion were conducted on limited patient populations and utilized retrospective methodologies. Therefore, caution should be exercised when interpreting these findings. Nonetheless, these studies provide valuable insights into potential prognostic factors for NHL, which can aid in the development of more effective treatment plans.

Elevation of LDH is a valuable biomarker and is associated with a higher tumor burden and more aggressive behavior of NHL, as well as an increased risk of central nervous system (CNS) relapse [26,27]. LDH is used in the IPI system and other prognostic nomogram models for aggressive B-cell lymphomas, as well as in other malignancies such as melanoma, prostate, and renal cell carcinomas as an independent prognostic factor [27]. Compared to late-relapsed, higher LDH levels, IPI scores, and stages (III-IV) were observed in early-relapsed cases. Huang's study, for instance, showed a higher relapse rate in pediatric patients with mature B-cell lymphoma, in whom LDH levels were >1,000 U/L [28]. Our study also found low survival rates in the morphological subtype of Mantle Cell and Nodal marginal zone lymphoma, and younger age, early stage, absence of B symptoms, non-elevated LDH, underwent radiotherapy, and complete remission was associated with higher survival rates [7,24,29].

The efficacy of autologous hematopoietic cell transplant with consolidation chemotherapy in relapsed NHL cases that responded to chemotherapy was evaluated in the PARMA trial, which showed a substantially higher rate of five-year event-free survival (46% vs. 12%, P = 0.001) and OS (53% vs. 32%, P = 0.038). Furthermore, the addition of rituximab to second-line chemotherapy has been shown to increase full response rates, as compared to second-line chemotherapy without rituximab [30]. However, in the present study, data regarding autologous hematopoietic cell transplants were not available for all patients, as some were referred to other centers.

Our study has several limitations that we need to acknowledge. Firstly, the sample size we collected from a single center in Najran City was small, which restricted the number of patients studied to 43. Secondly, the pathologic classification was done using the Working Formulation Group criteria, which is considered less accurate than newer criteria, but we did use immunohistochemical staining to determine the B-cell/T-cell subtype in all cases. Moreover, our pathologists were aware of the emergence of MALT lymphoma during the study period, which is now considered low-grade, and we excluded such cases from our study. Thus, we believe that our results are not outdated. Lastly, we could not include some relevant factors, such as predisposing factors and industrial exposures, in our analysis due to the nature of our study. To address these limitations and increase the validity and generalizability of our findings, future studies with larger sample sizes and prospective designs are recommended.

## Conclusions

This study is a retrospective single-center study that examines the distribution of the major NHL subtypes in Najran City. It provides a review of the existing understanding of the epidemiologic picture over a specific time period and offers a target for future research. The findings were comparable to those from other countries in terms of age, prevalence of common NHL subtype, and cancer stage. Our findings indicate a fluctuating trend of NHL, with a majority of cases being diagnosed at an advanced stage and among middle-aged individuals. Furthermore, our analysis demonstrated that patients with Burkitt lymphoma subtypes and elevated LDH had lower survival rates.
